# Expression of Tropomyosin 1 Gene Isoforms in Human Breast Cancer Cell Lines

**DOI:** 10.1155/2015/859427

**Published:** 2015-06-11

**Authors:** Syamalima Dube, Santhi Yalamanchili, Joseph Lachant, Lynn Abbott, Patricia Benz, Charles Mitschow, Dipak K. Dube, Bernard J. Poiesz

**Affiliations:** Division of Hematology/Oncology, Department of Medicine, SUNY Upstate Medical University, Syracuse, NY 13210, USA

## Abstract

Nine malignant breast epithelial cell lines and 3 normal breast cell lines were examined for stress fiber formation and expression of TPM1 isoform-specific RNAs and proteins. Stress fiber formation was strong (++++) in the normal cell lines and varied among the malignant cell lines (negative to +++). Although TPM1*γ* and TPM1*δ* were the dominant transcripts of *TPM1*, there was no clear evidence for TPM1*δ* protein expression. Four novel human TPM1 gene RNA isoforms were discovered (*λ*, *μ*, *ν*, and *ξ*), which were not identified in adult and fetal human cardiac tissues. TPM1*λ* was the most frequent isoform expressed in the malignant breast cell lines, and it was absent in normal breast epithelial cell lines. By western blotting, we were unable to distinguish between TPM1*γ*, *λ*, and *ν* protein expression, which were the only TPM1 gene protein isoforms potentially expressed. Some malignant cell lines demonstrated increased or decreased expression of these isoforms relative to the normal breast cell lines. Stress fiber formation did not correlate with TPM1*γ* RNA expression but significantly and inversely correlated with TPM1*δ* and TPM1*λ* expression, respectively. The exact differences in expression of these novel isoforms and their functional properties in breast epithelial cells will require further study.

## 1. Introduction

**Figure 1:**
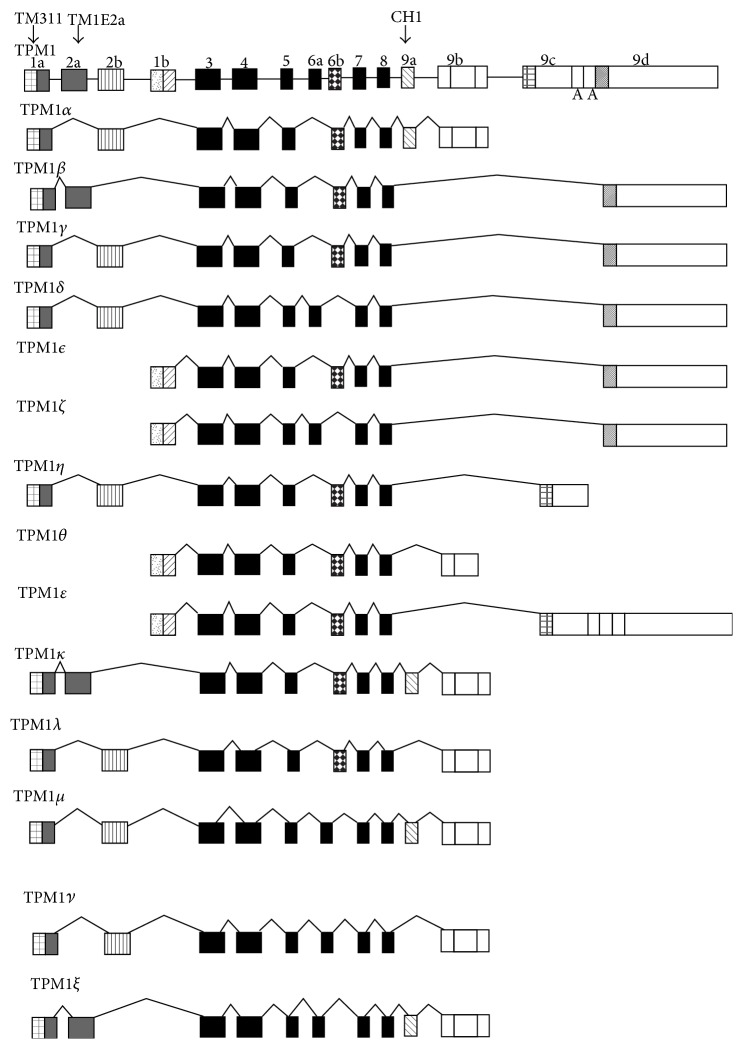
Cartoon showing the various exons (boxes) and introns (lines) of the human tropomyosin 1 gene and its various RNA isoforms identified to date. TPM1 *λ*–*ξ* are the newly described isoforms herein. The exons identified by the antibodies TM311, TM1E2*α*, and CH1 are identified. Those exons that are translated as peptides are indicated by solid or hatched, and so forth, markings, while those that are not are left blank. The translation profiles for TPM1 *λ*–*ξ* are assumed.

There are four relatively homologous human tropomyosin (TPM) genes which exhibit varying degrees of expression in human tissues [1–6]. Expression has been shown to be modulated at both the transcriptional and the translational levels [7, 8]. Multiple molecular TPM isoforms have been identified which are the results of alternative promoters or alternative splicing. Heretofore, there have been 10 isoforms (*α*–*κ*) of the TPM1 gene shown to be expressed in human tissues (Figure 1) [9]. TPM1*α* and *κ* have both been shown to be expressed in human cardiac tissue [10]. The former is expressed in greater amounts than the latter and, while both are incorporated into human cardiomyocyte myofibrils and are essential for myofibril formation, they have different biochemical properties [11, 12]. TPM1*κ* expression has been shown to be increased in the cardiomyocytes of patients with cardiomyopathy, although it is unknown whether this is a cause or an effect of the disease [12]. In a rat model, overexpression of human TPM1*κ* does cause cardiomyopathy [12, 13].

Tropomyosin proteins have been shown to make up some of the stress fibers of human epithelial cells and differences in their expression have been demonstrated in malignant breast epithelial cell lines and tissues compared to “normal” breast cell lines and tissues [1, 3, 14–20]. Much of this work has focused on the expression of TPM2*β* (also known as TM1). TPM1 *γ* and *δ* proteins (also known as *α*-tropomyosins) are known to be incorporated into epithelial cell stress fibers. It has been suggested that their expression may be downmodulated in human breast cancers [14–21]. However, because some novel tropomyosin 1 gene isoforms have been predicted (GenBank accession numbers: XP_005254697, XP_005254698, and XP_006720730) and identified in rat tissues, we decided to explore this issue further [22]. Hence, we examined stress fiber formation and TPM1 gene RNA and protein isoform expression in nine malignant and three benign human breast cell lines. Two human B-lymphocytic cell lines from two of the same breast cancer patients and human adult and fetal cardiac tissue were used as controls.

## 2. Materials and Methods

Human adult and fetal cardiac tissue RNA were obtained from Zyagen (San Diego, CA); similar protein samples were obtained from Imgenex (San Diego, CA). Human skeletal muscle proteins were obtained from Imgenex. The nonmalignant human breast cell lines MCF 10A, MCF 12A, and MCF 184B5, the normal human B-cell lines HCC 1143 (BL) and HCC 187 (BL), and the malignant human breast cancer cell lines HCC 1143, HCC 1187, BT 474, mDAMB 157, HCC 1806, HCC 1419, mDAMB 453, mDAMB 468, and MCF7 were obtained from ATCC (Manassas, VA).

## 3. Cell Culture

MCF7 were cultured in Eagles Minimum Essential Medium (ATCC) with 0.01 mg/mL bovine insulin (Sigma-Aldrich, St. Louis, MO) and 10% Fetal Bovine Serum (FBS) at 37°C in 5% CO_2_. MCF10A cells and 184B5 cells were cultured in Clonetics Mammary Epithelial Basal Medium (Lonza, Rochester, NY) with 100 ng/mL (MCF10A) or 1 ng/mL (184B5) cholera toxin (Sigma-Aldrich) at 37°C in 5% CO_2_. MDAMB468, MDAMB453, and MDAMB157 cells were cultured in Leibovitz's L-15 Medium with 10% FBS at 37°C in atmospheric air.

Cells were cultured in Corning T-75 flasks until 60–80% confluence and were then trypsinized and washed and 2-3 × 10^5^ cells were resuspended in 3 mLs of the appropriate culture medium and plated in MatTek Glass Bottom Culture Dishes (Day 0). Plates were incubated at 37°C (5% CO_2_ or atmospheric air) for 2 days and paraformaldehyde fixed for staining (Day 2). The cell lines were similarly cultured and cells were harvested for RNA and protein extraction.

## 4. Immunofluorescence

Cell cultures were fixed in 3% paraformaldehyde in PBS (phosphate buffered saline) for 15 minutes at room temperature. The fixed cells were rinsed twice with standard salt solution (0.1 M KCl), 0.001 M MgCl_2_, and 0.01 M phosphate buffer, pH.7.0, and permeabilized with 0.1% IGEPAL (Sigma-Aldrich) in standard salt for 10 minutes at room temperature. The free aldehyde groups in IGEPAL-treated cultured cells were removed by quenching with 50 mM NH_4_Cl for 5 min. Cells were then washed with salt solution for two minutes. The rinsing process was repeated 3 times and the cells were blocked with 1% BSA in standard salt for 1 hour. The blocked cells were rinsed for two minutes with standard salt. The cells were then incubated for 2 hours at room temperature with primary anti-tropomyosin antibody TM311 (1 : 1000 dilution) (Abcam, Cambridge, MA). Cells were washed eight times with standard salt and washed cells were incubated with Alexa Fluor 488 secondary antibody (Donkey Anti-Mouse IgG H&L (Abcam)) in 1 : 20 dilution for 1 hour at 37°C. The cells were washed eight times each for 3 min with standard salt. The cells were next stained with Alexa Fluor 594 phalloidin (diluted stock 1 : 25) (Life Technologies, Grand Island, NY) for 30 mins at room temperature and rinsed with standard salt (eight times) and distilled water for 5 mins ×3. Nuclei were then stained with DNA-binding DAPI [2- (4-Amidinophenyl) -6-indolecarbamide dihydrochloride] (Sigma-Aldrich) for 30 mins and rinsed 3 times with standard salt. Cells were mounted in Mowiol (Sigma-Aldrich) with 2.5% G n-Propyl Gallate (Sigma-Aldrich). The specimens were sequentially activated with different wave lengths of light and images were collected with a Leica AF6000 Deconvolution Microscope. The images were then merged to make a composite picture.

## 5. RNA and Protein Analyses

Total cellular RNA and protein were prepared from the samples, as previously described [24]. For RT-PCR, 0.5 *μ*g of RNA in a total volume of 40 mL was used to synthesize cDNA with SuperScript^R^II (Life Technologies, Grand Island, NY) and oligo-dT primers following the manufacturer's specifications. For each PCR 3 *μ*L of cDNA was used and GAPDH house-keeping gene RNA and TPM1 RNA were amplified as previously described [24]. Amplified cDNA was detected using Southern blot hybridization, as previously described [9]. Qualitative relative amounts of signal intensity were scored as negative up to ++++. The primer pairs and probes utilized are listed in Table 1. TPM1 RNA was amplified with several different primer pairs utilizing TPM1.Exon1a(+) as the plus primer and TPM1.Exon9a(−) (probe/prim), TPM1.Exon9b(−), or TPM1.Exon9d(−) as the negative primers. The amplified DNA was detected using the TPM1 generic probe TPM1.Exon3 (probe). TPM1 RNA was also amplified with several different primer pairs utilizing TPM1.Exon1b(+) as the plus primer and TPM1.Exon9b(−), TPM1.Exon9c(−), or TPM1.Exon9d(−) as the negative primers. The amplified DNA was detected using the TPM1 generic probe TPM1.Exon3 (probe). The amplified DNA was cloned and sequenced as described below. Upon obtaining sequence data, the amplified DNAs were repeatedly reexamined using the remaining more isoform-specific probes in Table 1. By using different combinations of primers and probes, a specific TPM1 isoform was identified and qualitatively quantified.

Amplified products were ligated and cloned into the TA cloning vector (Life Technologies) following our published protocol [25]. Positive clones were identified using the above mentioned specific probes, as previously described. Vectors or constructs were grown in* E. coli*, and the DNA was extracted using Qiagen mini-prep kit (Valencia, CA). The isolated DNA was sequenced (Cornell University Life Science Core Laboratories Center, Ithaca, NY). Each clone was sequenced twice in both directions.

In order to more accurately relatively quantify novel TPM1 isoforms found herein, we sequenced 14 colonies of cloned DNA amplified from each of the cell lines with the TPM1.Exon1a(+) and TPM1.Exon9b(−) primer pair. The relative frequency of each isoform in each sample was then calculated.

Protein from 10^6^ cells of each cell line was extracted with 100 *μ*L of cell extraction buffer (Life Technologies) containing 10 mM Tris, pH 7.4; 100 mM NaCl; 1 mM EDTA; 1 mM EGTA; 1 mM NaF; 20 mM Na_4_P_2_O_7_; 2 mM Na_3_VO_4_; 1% Triton X-100; 10% glycerol; 0.1% SDS; 0.5% deoxycholate. The cell extraction buffer was supplemented with 1 mM PMSF and protease inhibitor cocktail (Roche Diagnostics Corporation, Indianapolis, IN) following manufacturer specifications. The pellets were discarded and 10 *μ*L of supernatant from each sample was used for subsequent western blot analyses following our published protocol [26]. Primary antibodies included TM311 (Sigma-Aldrich, St. Louis, MO), anti-TPM1k [12], and CH1 anti-sarcomeric tropomyosin (Hybridoma Bank, DHSB, University of Iowa, Iowa). Secondary antibodies were goat anti-rabbit immunoglobulin HRP and sheep anti-mouse immunoglobulin HRP (GE Healthcare Bio-Sciences, Pittsburgh, PA). Results were scored as either negative or + to ++++. The correlation coefficients (*r*) between TPM1*γ*, TPM1*δ*, or TPM1*λ* RNA and stress fiber formation were calculated and plotted using Microsoft Excel. The *p* values were calculated using the Pearson calculator.

## 6. Results

**Figure 2:**
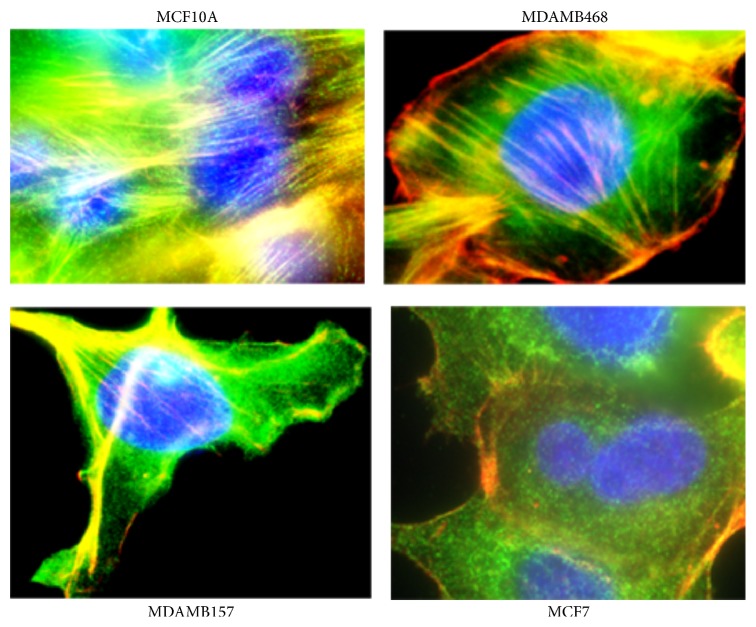
Merged photograph of one “normal” (MCF10A) and three malignant (MDAMB468, MDAMB157, and MCF7) human breast cell lines after staining with TM311, phalloidin, and DAPI. The stress fibers are the linear lines that contain both actin and tropomyosin resulting in a variety of colors depending on the background, while the nuclei are stained blue with DAPI. The cell lines were given the following stress fiber scores: MCF10A ++++, MDAMB468 +++, MDAMB157 ++, and MCF7 negative.

**Figure 3:**
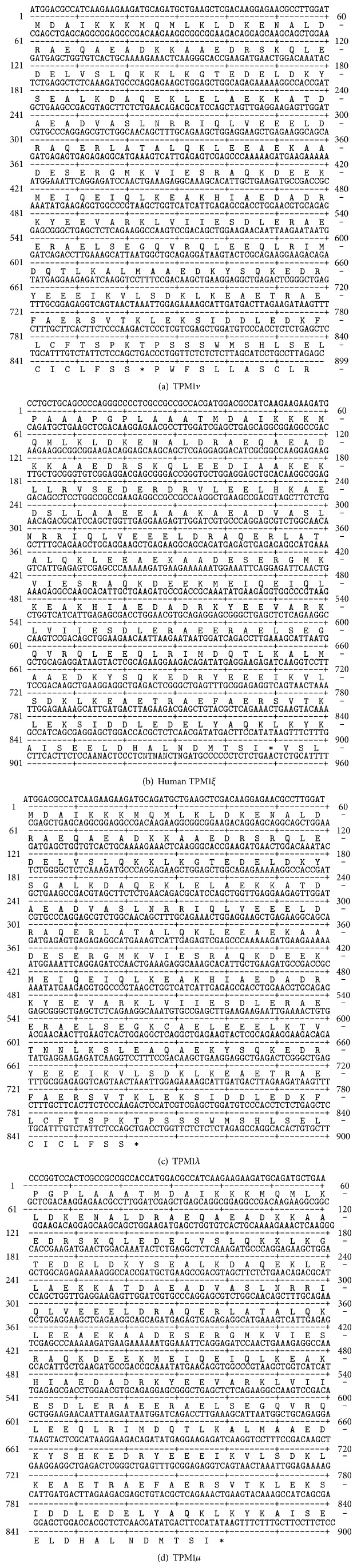
cDNA sequences (upper line) and amino acid translations (lower line) of TPM1 *λ*–*ε*. The symbol *∗* represents a stop codon.

**Figure 4:**
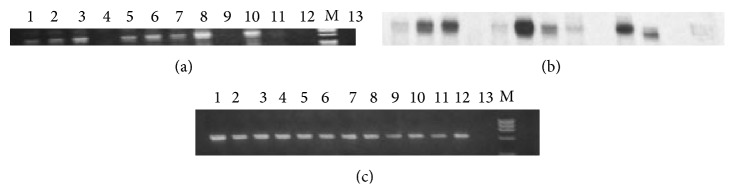
Expression of TPM1B transcripts in breast epithelial cell lines. cDNA from each cell line was amplified with the TPM1. Exon 1a(+)/TPM1. Exon 9d(−) primer pair (Table 1). Amplified DNAs were run on an agarose gel (Panel (a)) and subsequent hybridization was carried out using TPM1 exon 2a-specific 32P-labeled oligonucleotide probe (Panel (b)). Panel (c) depicts the ethidium bromide staining of the DNA amplified from human GAPDH cDNAs from each of the cell lines. Lane 1, MCF7; lane 2, MCF10A; lane 3, HCC1143; lane 4, HCC1143BL; lane 5, MDAMB453; lane 6, MDAMB468; lane 7, BT474; lane 8, HCC1187; lane 9, HCC1806; lane 10, MDAMB157; lane 11, HCC1419; lane 12, MDAMB1187BL; lane M, DNA marker; lane 13, primer only control.

**Figure 5:**
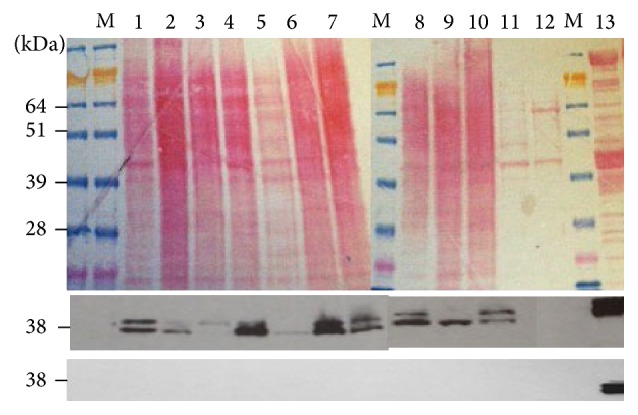
Western blot analyses for tropomyosin protein expressed in various breast epithelial cell lines. Top panel is the western blot stained with Ponceau dye. Middle panel is the blot stained with anti-tropomyosin antibody TM311 that reacts with all tropomyosins containing exon 1a. Bottom panel is the western blot stained with anti-tropomyosin antibody CH1 that reacts with all tropomyosins containing exon 9a. Lane M, molecular weight marker; lane 1, MDAMB453; lane 2, HCC1419; lane 3, HCC1806; lane 4, MDAMB157; lane 5, BT474; lane 6, HCC1187; lane 7, HCC1143; lane 8, MDAMB468; lane 9, MCF7; lane 10, MCF10A; lane 11, HCC1143BL; lane 12 HCC1187BL; lane 13, skeletal muscle.

Stress fibers containing both tropomyosin and actin filaments were identified in the cell lines to varying degrees ranging from negative to ++++ (Figure 2 and Table 2). Four new human TPM1 gene RNA isoforms (*λ*–*ξ*) were discovered in the breast epithelial cell lines (Figure 1) (Table 3). Their sequences and putative translation products are shown in Figures 2(a)–2(d). Three of these novel RNA sequences are identical to previously predicted variant TPM1 isoforms, while one, TPM1*ξ*, had not been predicted. However, several other predicted isoforms were not found. Figure 2 shows that both TPM1*λ* and TPM1*ν* encode proteins with 287 amino acid residues, whereas TPM1*μ* and TPM1*ξ* encode proteins with 284 amino acid residues. Interestingly, both TPM1*λ* and TPM1*ν* transcripts do not have exon 9a but do have exon 9b. This eliminates a stop codon and encodes for an additional 16 amino acids that would not be present in any other TPM1 isoform other than TPM1*θ* (Figure 3). TPM1*λ* and TPM1*ν* are differentiated by the fact that the former has exon 2b and exon 6b, but the latter contains exon 2a and exon 6a (Figure 1). Both TPM1*μ* and TPM1*ε* contain exon 9a and exon 9b. The protein coding ends at exon 9a and the stop codon as well as 3′-UTR comes from exon 9b. The only differences between these two isoforms are in exon 6. TPM1*μ* contains exon 6a whereas TPM1*ε* has got exon 6b (Figure 1). None of these novel isoforms were identified in the human B-cell lines or cardiac tissue. The B-cell lines were negative for any TPM1 gene expression and the human cardiac tissue contained only TPM1*α* and TPM1*κ* RNA (Tables 1 and 3), which were not found in the breast cell lines. Some TPM1*β* RNA expression but not protein expression was detected (Figures 4 and 5 and Tables 3 and 4) in breast epithelial cell lines. TPM1*γ* and TPM1*δ* were the dominant RNA isoforms expressed, although no evidence for TPM1*δ* protein expression could be identified (Tables 3 and 4). However, we had no definite positive control for TPM1*δ* protein. The skeletal muscle 34 kD protein detected with TM311 is probably TPM3*α*. It is conceivable that TPM1*γ* and *δ* are running together in the 36 kD band. TPM1*λ* RNA was identified in most malignant breast cancer cell lines but not in the benign cell lines, while TPM1*ξ* RNA was identified only in the MCF 10A cell line (Table 3). There was no evidence for TPM1*μ* and TPM1*ξ* protein expression (Table 4). TPM1*λ* and *ν* would only be detected by the TM311 antibody. However, because we do not know their exact size on SDS PAGE, we cannot speculate as to whether they were expressed or not. We would assume that they could be very close in size to TPM1*γ*. However, because TPM1*λ* has a few more amino acids than TPM1*γ*, it could be slightly higher on an SDS PAGE gel. As can be seen in Table 4, it is also possible there is non-TPM1, TPM isoform protein in the breast epithelial lines, which presumably would be products of the TPM2 and/or TPM4 genes.

**Figure 6:**
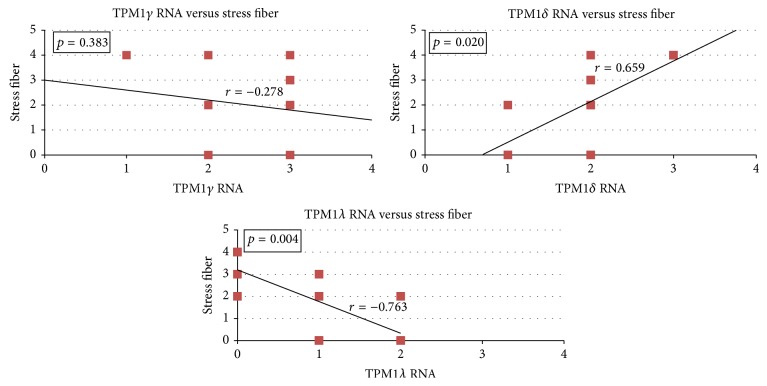
Regression analyses of stress fiber formation versus expression of TPM1*γ*, TPM1*δ*, or TPM1*λ* RNA among the breast epithelial cell lines. Each box represents one or more cell line(s). The correlation coefficients (*r*) and *p* values for each analysis are shown. As can be seen, TPM1*γ* expression did not have a high correlation with stress fiber formation, while TPM1*δ* and TPM1*λ* RNA expression had high, significant, positive, and inverse correlations with stress fiber formation.

Figure 6 shows the relationship between stress fiber formation and TPM1*γ*, TPM1*δ*, or TPM1*λ* RNA expression among the normal and malignant breast cell lines. There was no significant correlation between TPM1*γ* RNA and stress fiber formation, while TPM1*δ* RNA expression and TPM1*λ* RNA expression demonstrated significant positive and inverse correlations, respectively, with stress fiber formation.

## 7. Discussion

The actin cytoskeleton in epithelial cells contains stress fibers comprised of actin microfilaments and various actin-binding proteins [27]. They play a major role in anchorage dependence, cell locomotion, and proliferation. Their dysregulation is felt to play a role in cellular transformation and metastasis [14, 16, 28, 29]. Various isoforms of tropomyosin play a critical role in the normal function of these microfilaments and alterations in their expression are felt to be involved in oncogenesis.

The high molecular weight tropomyosin isoforms TPM1*γ* and *δ* and TPM2*β* have been shown to be expressed in normal human breast epithelial cells and their protein products are incorporated into stress fibers [14]. The lower molecular weight isoforms TPM1*ε* and TPM4*τ* have also been shown to be expressed [14]. Downregulation of TPM1*γ* and *δ* and TPM2*β* isoforms has been observed in some but not all malignant human breast cell lines and primary breast carcinomas [14, 16, 28, 29]. Paradoxically, levels of TPM2*β* were found to be elevated in primary breast cancers that gave rise to lymph node metastases compared to those that did not [14]. The data presented herein indicate that expression of TPM1*δ* but not TPM1*γ* RNA had a positive correlation with stress fiber formation in the cell lines studied.

Given the identification and/or prediction of an increasingly greater number of tropomyosin isoforms than known in the past, we have embarked on a systematic examination of various human cell types for novel isoform expression. Indeed, in the data presented herein, we have detected four TPM1 RNA isoforms not identified in human tissues before. With the reagents and one-dimensional SDS PAGE techniques utilized we are unable to say whether TPM1*λ* and *ν* are expressed as proteins or not. We suspect that they could be running with TPM1*γ* on the gel. In fact, we were not able to detect TPM1*δ*, which others have shown to be expressed in human breast epithelial cells [14]. We also suspect it too may be overlapping with TPM1*γ* on our gels. These uncertainties should be able to be resolved with further studies employing a 2D SDS PAGE approach or by developing antibodies against the unique amino acids of the carboxy terminus of TPM1*λ* and *ν*.

At the RNA level, there were varying degrees of expression of the novel TPM1 isoforms between the “normal” and malignant breast epithelial cell lines. Of particular interest was the fact that TPM1*λ* was upregulated in many of the malignant cell lines compared to the normal cell lines. This increased RNA expression had a strong, statistically significant, inverse correlation with stress fiber formation among the cell lines. Again, future studies are required to study these differences at the protein level and whether they can be detected in fresh normal and malignant breast epithelial cells. Structural studies examining the incorporation into stress fibers and cellular location of the novel TPM1 isoforms and functional studies regarding the impact of their over- and underexpression are also warranted.

## 8. Conclusions

Four novel tropomyosin RNA isoforms were detected in human breast epithelial cell lines, with varying degrees of expression between benign and malignant cells. Their function remains to be determined. TPM1*δ* and TPM1*λ* RNA had significant positive and inverse correlations, respectively, with stress fiber formation among the cell lines. The data would indicate that tropomyosin control of breast epithelial growth may be more complex than previously thought.

## Figures and Tables

**Table 1:** Nucleotide sequences of the primer pairs and probes used to amplify and detect TPM2 RNAs.

TPM1.Exon 1a (+): 5′-GCTCCTGCTGCAGCCCCAGG-3′	
TPM1.Exon 9b (−): 5′-CTTCTGTACAATAGAAAGCA-3′	
TPM1.Exon 9d (−): 5′-TTCACATGTTGTTTAACTCCAGT-3′	

TPM1.Exon 2a (probe): 5′-GAA GTT GCT GCG GGT GTC GG-3′	
TPM1.Exon2b (probe): 5′-AAG ATG AAC TGG ACA AAT AC-3′	
TPM1.Exon 3 (probe): 5′-ATCGTGCCCAGGAGCGTCTGGCAA-3′	
TPM1.Exon 6a (probe): 5′-GAAAGCATTAATGGCTGCAGAGG-3′	
TPM1.Exon 6b (probe): 5′-TGAAGTCACTGGAGGCTCAGG-3′	
TPM1.Exon 9a (−) (probe/prim) 5′-ATGTCGACCTTATATGGAAGTCATATCG-3′	

GAPDH. P1(+): 5′-GTTTACATGTTCCAATATGATTCCAC-3′	
GAPDH.P2(−): 5′-TCATATTTGGCAGGTTTTCTAGA-3′	
GAPDH.Probe: 5′-GTGGAGTCCACTGGCGTCTT-3′	

TPM2.Exon 1a(+): 5′-ATGGACGCCATCAAGAAGAA-3′	
TPM2.Exon 9d(−): 5′-TGGGGCTGGCCCTCACAGGTT-3′	
TPM2.Probe: 5′-AGAGGGCTGAGGTGGCCGAGAGCCG-3′	

**Table 2:** Tropomyosin 1 gene RNA isoform expression in breast cancer cell lines.

Cell line	RNA isoform expression^▲^														
	TMP1*α*	TPM1*β*	TPM1*γ*	TPM1*δ*	TMP1*ε*	TPM1*ζ*	TPM1*η*	TPM1*θ*	TPM1*ι*	TPM1*κ*	TPM1*λ*	TPM1*μ*	TPM1*ν*	TPM1*ξ*	GAPDH
Breast cancer															
HCC 1143^*∗*^	−	+	+++	++	−	−	−	−	−	−	−	+	+	−	++++
HCC 1187^*∗*^	−	+	+++	++	−	−	−	−	−	−	++	+	−	−	++++
BT 474	−	+	+++	++	−	−	−	−	−	−	+	−	−	−	++++
mDAMB 157	−	+	+++	++	−	−	−	−	−	−	+	+	++	−	++++
HCC 1806	−	−	++	+	−	−	−	−	−	−	−	+	+	−	++++
HCC 1419^*∗*^	−	+	++	++	−	−	−	−	−	−	+	−	+	−	++++
mDAMB 453	−	−	++	+	−	−	−	−	−	−	++	−	+	−	++++
mDAMB 468	−	+	+++	++	−	−	−	−	−	−	+	+	+	−	++++
MCF7	−	−	+++	+	−	−	−	−	−	−	++	−	−	−	++++

MCF10A (normal breast)	−	+	+++	++	−	−	−	−	−	−	−	+	+	+	++++
HCC 1143 (BL)^+^	−	−	−	−	−	−	−	−	−	−	−	−	−	−	++++
HCC 1143 (BL)^+^	−	−	−	−	−	−	−	−	−	−	−	−	−	−	++++
Adult heart	++++	−	−	−	−	−	−	−	−	+	−	−	−	−	++++
Fetal heart	+++	−	−	−	−	−	−	−	−	++	−	−	−	−	++++

▲ = cell lines were analyzed by RNA-PCR for the indicated TPM1 isoforms. GAPDH RNA was analyzed as a control for baseline RNA expression. Expression was qualitatively recorded as either negative (−) or plus (+) to four pluses (++++).

*∗* = primary tumor; the rest were metastatic tumors.

+BL = B-lymphocyte cell lines.

**Table 3:** Tropomyosin 1 RNA isoform frequencies in various human cardiac tissues and breast epithelial cell lines.

Sample	Tropomyosin 1 RNA isoform frequencies among cDNA clones					
	*α* (%)	*κ* (%)	*λ* (%)	*μ* (%)	*ν* (%)	*ξ* (%)
Fetal heart	69 (69)	31 (31)	0 (0)	0 (0)	0 (0)	0 (0)
Adult heart	16 (80)	4 (20)	0 (0)	0 (0)	0 (0)	0 (0)

Malignant breast cell lines						
HCC 1143	0 (0)	0 (0)	0 (0)	6 (43)	8 (57)	0 (0)
HCC 1187	0 (0)	0 (0)	9 (64)	5 (36)	0 (0)	0 (0)
BT 474	0 (0)	0 (0)	14 (100)	0 (0)	0 (0)	0 (0)
mDAMB 157	0 (0)	0 (0)	2 (14)	3 (22)	9 (64)	0 (0)
HCC 1806	0 (0)	0 (0)	0 (0)	1 (7)	13 (93)	0 (0)
HCC 1419	0 (0)	0 (0)	5 (36)	0 (0)	9 (64)	0 (0)
mDAMB 453	0 (0)	0 (0)	9 (64)	0 (0)	5 (36)	0 (0)
mDAMB 468	0 (0)	0 (0)	5 (36)	4 (28)	5 (36)	0 (0)
MCF 7	0 (0)	0 (0)	14 (100)	0 (0)	0 (0)	0 (0)
Normal breast cell line						
MCF 10	0 (0)	0 (0)	0 (0)	3 (22)	10 (71)	1 (7)

**Table 4:** Tropomyosin 1 protein expression in breast cancer cell lines.

Cell line	Antibody and TPM isoforms detected^▲^							
	TM311^Δ^						TPM1E2a	CH1
	>40 kD	40 kD	38 kD	36 kD	34 kD	<34 kD		
	TPM2*γ*	TPM1*β*, *κ*	TPM2*β*, *η*	TPM1*α*, *γ*	TPM1*δ*, *η*	TPM2*ε*, *ζ*	TPM1*β*, *κ*, *ξ*	TPM1*α*, *κ*, *μ*, *ξ*
								TPM2*α*
		TPM2*α*, *δ*	TPM4*δ*, *ε*		TMP3*α*			TPM3*α*, *δ*, *ε*, *θ*, *ι*, *κ*, *λ*, *μ*, *ν*
Breast cancer								
HCC 1143^*∗*^	−	−	++	++	−	−	−	−
HCC 1187^*∗*^	−	−	++	+++	−	−	−	−
BT 474	−	−	−	+	−	−	−	−
mDAMB 157	−	−	+	++++	−	−	−	−
HCC 1806	−	−	+	+	−	−	−	−
HCC 1419^*∗*^	−	−	+	+	−	−	−	−
mDAMB 453	−	−	++	++	−	−	−	−
mDAMB 468	−	−	++	+++	−	−	−	−
MCF7	−	−	++	++	−	−	−	−

MCF10A (normal breast)	−	−	+++	++	−	−	−	−
HCC 1143 (BL)^+^	−	−	−	−	−	−	−	−
HCC 1187 (BL)^+^	−	−	−	−	−	−	−	−

Adult heart	−	+	+	++++	−	−	+	++++
Fetal heart	−	+	+	++++	−	−	+	++++
Skeletal muscle	−	+++	−	+++	+++	−	−	++++

The symbols *∗* and + are as described in Table 1.

▲ = cell extracts were analyzed by western blot using the various primary antibodies indicated. Expression was qualitatively recorded as either negative (−) or plus (+) to 4 pluses (++++).

Δ = the assignations of various TPM protein isoforms to particular sizes on an SDS PAGE gel are as per Schevzov  et al. [23].

TPM1*λ*, *μ*, *ν*, and *ξ* would be detected by TM311, but we do not know their sizes on SDS PAGE.
